# Impaired LDL Receptor-Related Protein 1 Translocation Correlates with Improved Dyslipidemia and Atherosclerosis in apoE-Deficient Mice

**DOI:** 10.1371/journal.pone.0038330

**Published:** 2012-06-06

**Authors:** Philip L.S.M. Gordts, Alexander Bartelt, Stefan K. Nilsson, Wim Annaert, Christina Christoffersen, Lars Bo Nielsen, Joerg Heeren, Anton J.M. Roebroek

**Affiliations:** 1 Laboratory for Experimental Mouse Genetics, Center for Human Genetics, KU Leuven, Leuven, Belgium; 2 Department of Biochemistry and Molecular Cell Biology; 3 Department of Orthopedics University Medical Center Hamburg-Eppendorf, Hamburg, Germany; 4 Department of Medical Biosciences/Physiological Chemistry, Umeå University, Umeå, Sweden; 5 Laboratory of Membrane Trafficking, Department of Molecular and Developmental Genetics, VIB, Leuven, Belgium; 6 Laboratory of Membrane Trafficking, Center for Human Genetics, KU Leuven, Leuven, Belgium; 7 Department of Clinical Biochemistry, Rigshospitalet, Copenhagen, Denmark; 8 Department of Biomedical Sciences, University of Copenhagen, Copenhagen, Denmark; Baylor College of Medicine, United States of America

## Abstract

**Objective:**

Determination of the *in vivo* significance of LDL receptor-related protein 1 (LRP1) dysfunction on lipid metabolism and atherosclerosis development in absence of its main ligand apoE.

**Methods and Results:**

LRP1 knock-in mice carrying an inactivating mutation in the NPxYxxL motif were crossed with apoE-deficient mice. In the absence of apoE, relative to LRP1 wild-type animals, LRP1 mutated mice showed an increased clearance of postprandial lipids despite a compromised LRP1 endocytosis rate and inefficient insulin-mediated translocation of the receptor to the plasma membrane, likely due to inefficient slow recycling of the mutated receptor. Postprandial lipoprotein improvement was explained by increased hepatic clearance of triglyceride-rich remnant lipoproteins and accompanied by a compensatory 1.6-fold upregulation of LDLR expression in hepatocytes. One year-old apoE-deficient mice having the dysfunctional LRP1 revealed a 3-fold decrease in spontaneous atherosclerosis development and a 2-fold reduction in LDL-cholesterol levels.

**Conclusion:**

These findings demonstrate that the NPxYxxL motif in LRP1 is important for insulin-mediated translocation and slow perinuclear endosomal recycling. These LRP1 impairments correlated with reduced atherogenesis and cholesterol levels in apoE-deficient mice, likely via compensatory LDLR upregulation.

## Introduction

Cardiovascular diseases (CVD) are the leading cause of death in Western societies and are primarily caused by complications of atherosclerosis. The pathology is characterized by a thickening of the arterial wall, resulting in the narrowing of the lumen of arteries and consequently reducing the blood flow to critical levels in many vital organs. It is a very complex and still not completely elucidated process characterized by accumulation of lipids, inflammatory cells, and fibrous elements in large and medium-sized arteries [1]. According to the response-to-retention model, the initiating event in atherogenesis is focal infiltration and retention of apolipoprotein (apo) B containing lipoproteins, like Low-Density Lipoprotein (LDL), Lipoprotein(a), and triglyceride-rich remnant lipoproteins (TRLs) in the subendothelial matrix of arteries [2]. ApoE plays several critical roles in regulating plasma lipid and lipoprotein levels [3]. One of its functions is to serve as a ligand that mediates the binding, uptake, and plasma clearance of TRLs via cell surface receptors being the heparan sulfate proteoglycan (HSPG) syndecan 1, the LDL Receptor (LDLR), and LDLR-Related Protein 1 (LRP1) [4]–[9]. LRP1 is a large multifunctional receptor that binds many different ligands. LRP1 is proteolytically cleaved by furin into two subunits, one of 515 kDa, containing the extracellular binding domains (LRP1-α), and one of 85 kDa (LRP1-β), comprising the membrane spanning and cytoplasmatic domains [10]. Currently it is believed that hepatic LRP1 serves as a back-up receptor for LDLR in mediating TRL clearance as its absence in the liver in mice only affects triglyceride levels in LDLR deficiency [6]. However, hepatic clearance of postprandial generated TRLs is in part dependent on an insulin-mediated translocation of LRP1 from intracellular storage compartments to the plasma membrane (PM) [11]. Hepatic LRP1-deficient mice, lacking postprandial insulin-dependent LRP1 activity at the PM, showed attenuated TRL uptake despite presence of LDLR and HSPGs.

The defective binding of apoE to receptors involved in TLR clearance is essentially a major cause of hypertriglyceridemia or type III hyperlipidemia [12]. Type III hyperlipidemia is a genetic disorder characterized by the accumulation of TRLs in the plasma and premature atherosclerosis development. There are three common isoforms for apoE, all with different binding properties to LDLR. ApoE3 (Cys112 and Arg158) is the most common isoform in humans, second apoE4 (Arg112 and Arg158), and last apoE2 (Cys112 and Cys158). The primary molecular cause for type III hyperlipidemia is *APOE2* homozygosity as it is characterized by a very low binding affinity to the LDLR [3], [13]. However, only 10% of the *APOE2* homozygotes are hyperlipidemic, while the majority of the individuals display a balanced dyslipidemia and are normolipidemic or even hypocholesterolemic [12]. Importantly, normo- or even hypolipidemic *APOE2* homozygote patients have no increased risk for CVD [14]. The development of type III hyperlipidemia therefore requires apoE2 plus a secondary genetic or environmental factor.

It is possible that LRP1 dysfunction is a secondary factor contributing to the development of type III hyperlipidemia. Recent data from a genome-wide association study support this hypothesis, as they identified LRP1 as a risk factor for triglyceride levels [15] and *in vitro* studies showed that atorvastatin treatment resulted in up-regulation of hepatic LRP1, which might explain why statin treatment decreases TRLs [16]. Also concomitant LRP1 dysfunction could have an impact by multiple mechanisms on atherosclerosis development, which is more frequently observed in type III hyperlipidemia patients. The impact of LRP1 dysfunction on cardiovascular disease likely extends beyond effects on the lipoprotein metabolism, as apoE mediates its inhibitory signals on SMC migration in part via LRP1 [17] and because other signaling pathways involved in atherosclerosis, like Liver X Receptor-mediated gene transcription, are also regulated through LRP1 [18], [19] Recently, two studies showed that LRP1 is important for limiting macrophage apoptosis and inflammatory monocytosis in atherosclerotic lesions likely independent from apoE [20], [21].

In the present study, we investigated the *in vivo* impact of LRP1 dysfunction on lipid metabolism and atherosclerosis development in the absence of apoE. To tackle this question, we made use of knock-in mice expressing a dysfunctional LRP1 [22] crossed into the apoE^−/−^ background. The use of this mouse model allowed us to investigate the role of LRP1 independently of its role in the catabolism of apoE-rich lipoproteins.

## Materials and Methods

### Ethics Statement

Animal experiments were approved by the Institutional Animal Care and Research Advisory Committee of the KU Leuven.

### Animals

Homozygous LRP1 knock-in mutant mice (mixed C57Bl/6J and 129 background), containing the previously described NPxYxxL knock-in mutation (PTNFTNPVYATL PTNFTAAVAATL, LRP1^n2/n2^) [22], were crossed with homozygous apoE knock-out mice (apoE^−/−^) [23] on a C57BL/6J background (Jackson Laboratories, Bar Harbor, Maine, USA). The obtained apoE^−/−^LRP1^n2/n2^ and apoE^−/−^LRP1^+/+^ ( =  apoE^−/−^) mice (87.5% C57Bl/6J) and their offspring were used for further experimental analysis. Unless otherwise indicated, the experiments were performed on 12-weeks old mice. Prior to organ sampling, blood was removed by cardiac puncture and the animal was perfused through the left ventricle with 10 ml phosphate-buffered saline (PBS). Animals were maintained on a 12-h light, 12-h dark cycle and received tap water *ad libitum*.

### Serum Lipids and Lipoprotein Distribution Analysis

Serum samples were obtained by cardiac puncture from mice fasted for 16 hours, or via tail bleeding from 5 hours fasted mice using microvette CB 300 capillaries (Sarstedt, Nümbrecht Germany). Cholesterol and triglyceride levels were measured by commercially available enzymatic kits (Roche, Mannheim, Germany). Lipoprotein profiles were performed on pooled plasma from six mice per genotype. Lipoproteins of 200 µl pooled plasma samples were separated by fast performance liquid chromatography (FPLC) gel filtration on a Superose 6 column and cholesterol and triglycerides were determined in each fraction [24].

### Hepatic VLDL-Triglyceride Secretion and Intestinal Lipid Absorption

Mice were fasted for 5 h and anesthetized with an intraperitoneal injection (i.p.) of pentobarbital (70 µg/g body weight Nembutal, CEVA Santé Animale, Brussels, Belgium) prior to a tail vein injection of Tyloxapol (10% solution in PBS; Sigma-Aldrich, Bornem, Belgium) at a dose of 0.5 mg/g body weight. Plasma was collected by tail bleeding at time points 1, 15, 30, 60, and 120 min after injection. To measure intestinal lipid absorption, fasted mice received an intragastric load of 10 µl/g body weight of olive oil. One min later mice received an intravenous injection of Tyloxapol (0.5 mg/g body weight, 10% solution in PBS; Sigma-Aldrich, Bornem, Belgium) to block plasma lipoprotein clearance [25]. Blood samples (60 µl) were drawn before gavage (time 0) and 1, 2, and 3 hours after gavage by tail bleeding. Plasma TG was measured as described above.

### Postprandial Triglyceride Response

Mice were fasted for 5 h prior to receiving an intragastric load of olive oil (10 µl/g body weight). Plasma was collected by tail bleeding for triglyceride measurements at time points 0, 60, 120, 180, and 240 min after injection. Postprandial lipid accumulation in organs was evaluated as described [26]. Briefly, mice were fasted for 5 hours prior to receiving an intragastic fat load with 200 µl olive oil containing 40 µCi glycerol-tri-[1-^3^H]oleate (Amersham-Biosciences, Freiburg, Germany) via oral gavage. After 2 hours, blood was removed by cardiac puncture and the carcass was perfused with 10 ml of PBS containing 10 units of heparin before harvesting organs. For ^3^H measurements, organs were solubilized in Solvable (PerkinElmer, Waltham, USA) and counted in scintillation fluid.

### Cell Culture

The previously described LRP1 wild-type and NPxYxxL mutant mouse embryonic fibroblast (MEFs) [22] were used for comparative analysis. The cells were cultured in Dulbecco’s modified Eagle’s medium (DMEM) containing 10% fetal bovine serum (FBS; Invitrogen, Merelbeke, Belgium), 100 units/ml penicillin, and 0.1 mg/ml streptomycin at 37°C in an atmosphere of 5% CO2. Mouse hepatocytes were prepared from 8-week old mice by liver perfusion with EDTA dissociation followed by Percoll centrifugation as described [27]. Hepatocytes were cultured in DMEM containing 10% FBS, 100 units/ml penicillin, and 0.1 mg/ml streptomycin for 14 hours prior to *in vitro* experiments. Before insulin stimulation, cells were put overnight with DMEM containing 10% lipoprotein-deficient FBS (LPDS), 100 units/ml penicillin, and 0.1 mg/ml streptomycin and stimulated for 15 min with or without 100 nM insulin in DMEM containing 10% lipoprotein-deficient FBS (LPDS), 100 units/ml penicillin, and 0.1 mg/ml streptomycin.

### *In vitro* Binding and Uptake Experiments

The fluorescent endocytosis assay was adapted from the procedure performed by Dedieu et al [28] as previously described [29]. Briefly, MEFs were washed twice with PBS and incubated for 1 hour at 37°C for uptake, or at 4°C for binding in fresh serum-free medium containing 50 µg/ml FITC labelled human α_2_M (Biomac, Liepzig, Germany) alone, or together with 1 µM receptor associated protein (RAP), in the presence or absence of 100 µM chloroquine to inhibit lysosomal activity or MG132 to inhibit proteasomal degradation. Human RAP was expressed in bacteria as a fusion protein with glutathione S-transferase and was purified as described previously [30]. Uptake of chylomicron remnants (CR) was performed in hepatocytes as described by Niemeier et al. [31]. Chylomicrons (CM) were obtained from plasma of a non-fasted patient with an apo C-II deficiency by density ultracentrifugation and hydrolyzed *in vitro* to obtain CR [32]. For CR-K1 generation, healthy male individuals (23–38 years of age) carrying the apoE3/3 genotype underwent a standardized oral fat load in the form of a heavy breakfast (1950 kcal, 46% fat, 37% carbohydrates, and 17% protein), supplemented with an oral dose of 10 mg vitamin K1 [33]. These particles were isolated 4 hours postprandial by ultracentrifugation without further *in vitro* hydrolysis and designated CR-K1 (with supplementation of vitamin K1). The lipoproteins were labeled with ^125^I by the iodine monochloride method [32]. In brief, uptake experiments were performed in hepatocytes seeded into collagen coated 6-well plates (Nalge Nunc International, NY, USA) at 400 000 cells/well and treated for 16 hours with DMEM containing 10% LPDS. ^125^I-CR or ^125^I-CR-K1 were added in a concentration of 5 µg/ml in DMEM containing 10% LPDS. After incubation, the cells were washed four times with PBS. Surface bound CR was then released by PBS containing heparin. Hepatocytes were solubilized in 0.1 M NaOH. Finally, total radioactivity and total cell protein of the lysate were determined. All uptake data are obtained as triplicates.

### Cell Surface Fluorescence Quenching Recycling Assay

LRP1 wild-type and NPxYxxL mutant MEFs were used to measure LRP1 recycling as described [34], [35]. The monoclonal 5A6 antibody (Gentaur, Brussels, Belgium), recognizing the extracellular β-subunit of LRP1, was conjugated with Alexa488 using the Alexa fluor 488 Protein Labeling kit (Invitrogen, Merelbeke, Belgium) according to the manufacturer’s instructions. MEF cells were incubated with the 40 µg/ml Alexa488-labeled 5A6 antibody in warm binding buffer (DMEM containing 0.6 g/l BSA) for 20 to 30 min at 37°C. Following removal of fluorescent antibody in the medium, cells were incubated for indicated times in the absence or presence of 24 µg/ml of the quenching antibody anti-Alexa488 IgG in binding buffer at 37°C (Invitrogen, Merelbeke, Belgium). Since fluorescence of a recycled receptor is quenched by the anti-Alexa488 antibody at the cell surface, the reduction in total fluorescence represents a measure for recycling of the receptor. Percentage of the initial fluorescence (pulse) remaining at each time point was calculated as the difference between non-chased (time 0) and chased cell fluorescence.

### Cell Fractionation Experiments

Cell fractionation was carried out as described previously [29]. All steps of the fractionation protocol were carried out at 4°C. For each fraction, equal volumes (83 µl) of protein were used for further processing by immunoblot analysis.

### En Face Quantification of Aortic Lesions

Mice euthanized by CO_2_ intoxication were perfused with 10 ml PBS followed by 20 ml freshly prepared paraformaldehyde (PFA) (4% [wt/vol] in PBS). The heart and ascending aorta down to the iliac bifurcation were removed and incubated in PFA. The heart and adventitial tissue were removed, the aortas were cut open, pinned flat and stained for neutral lipids using Sudan IV. Images were acquired using a Leica IC A video camera attached to a Leica MZ FLIII stereomicroscope and lesions from blinded samples were measured using image analysis software Leica IM1000 (Leica Microsystems Ltd, Heerbrugg, Switzerland). The data are expressed as the percentage of the total Sudan IV positive lesion areas compared to the total aortic area.

### Immunofluorescence on Primary Hepatocytes

Hepatocytes were plated on collagen coated glass coverslips placed in 24-well plates at 100, 000 cells/well for 16 hours. Cells were stained for immunofluorescence as previously described [11]. The antibody recognizing LDLR is from Progen and the anti-LRP1 polyclonal antibody 377-4 was kindly provided by Prof. J. Herz, Dallas, TX. Cell images were taken with an Axiovert 100 microscope equipped with a Zeiss Axiocam. Confocal images were collected using a Zeiss LSM 510 (version 3.0).

### Immunoblot Analysis

Cell homogenates were made using ice-cold lysis buffer (50 mM NaF, 1 mM Na_2_.EDTA, 1 mM EGTA, 20 µM phenylarsine oxide, 5 mM Na_3_VO_4_, 1% Triton X-100, and proteinase inhibitors [Complete™, Roche Applied Science GmbH, Mannheim, Germany]). Cell homogenates were centrifuged for 20 min at 15000×*g* and the supernatant was collected. Liver tissue was homogenized with a motor Potter-Elvehjem tissue grinder (Wheaton) in 250 mM sucrose, 10 mM Tris-HCl (pH 7.4) and proteinase inhibitors [Complete™]. Postnuclear supernatant of liver tissue was centrifuged for 1 hour at 100,000×*g* to obtain a soluble microsomal fraction. The pellet containing the microsomal fraction was dissolved in ice-cold lysis buffer. After being fasted overnight mice received an i.p. insulin (1 U per kg) or NaCl injection before being sacrificed 5 min later for liver extraction and preparation of purified PM as described [36]. Equal amounts of homogenates, postnuclear supernatant, microsomal fraction or purified PM were run on SDS-PAGE 4–12% Bis-Tris NuPage (Invitrogen, Merelbeke, Belgium) gels as previously described [22]. The 1704 antibody (kindly provided by Prof. C. Pietrzik, Mainz, Germany) was used to recognize the LRP1 C-terminus. Anti-apolipoprotein AI, B and E, anti-LDLR and anti-β-actin antibodies were purchased from respectively Abcam, Labconsult, Acris, Abcam and Sigma-Aldrich. Images were analyzed with NIH image software (Image J, 1.44e; NIH, Bethesda, Maryland).

### Statistical Analysis

Statistical significance between groups was determined by Student́s *T-test* and Mann-Whitney rank sum tests. Correlations were calculated by Pearson’s correlation coefficient (Rp) using STATISTICA version 6 software (StatSoft Inc). *P*<0.05 was regarded as statistically significant.

## Results

### Inactivation of the LRP1 NPxYxxL Motif Causes Accelerated Postprandial Lipid Clearance

**Figure 1:**
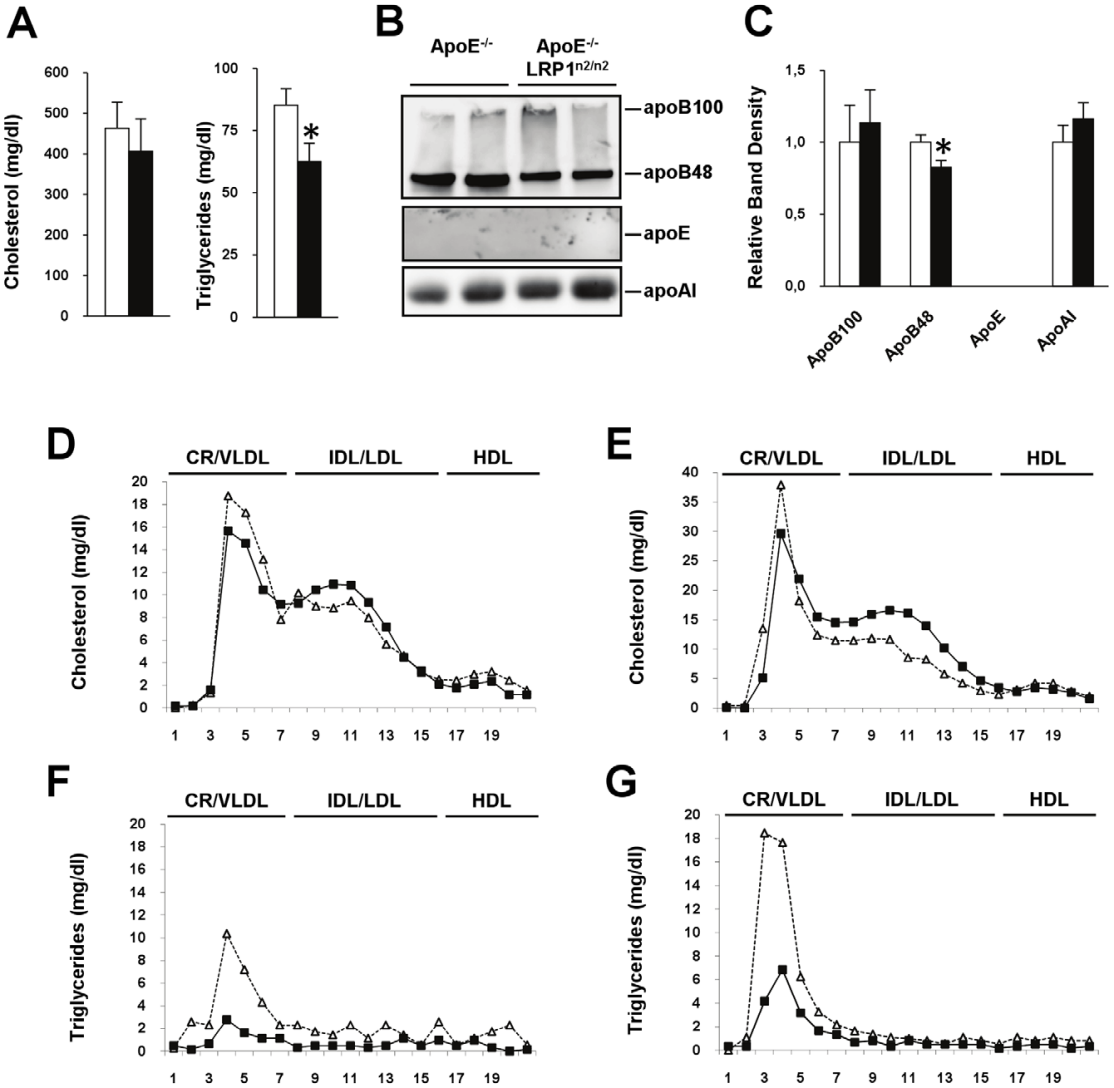
Analyses of total cholesterol, triglycerides and lipoprotein profiles in ApoE^−/−^ (open bars or Δ) and ApoE^−/−^LRP1^n2/n2^ (filled bars or ▪) mice. A–C, Plasma lipid levels (A), immunoblot analysis of plasma apolipoproteins (B) and their relative expression levels (C) (n = 8 per genotype). D–G, Lipoprotein profiles in fasted and postprandial state (pooled plasma from six mice per genotype). Plasma lipoprotein distribution of cholesterol (D–E) and triglyceride (F–G) levels in 5 hour fasted apoE^−/−^ and apoE^−/−^LRP1^n2/n2^ mice just before (D & F) or 2 hours after receiving a gastric olive oil load (E & G). Data are mean±SEM. *P<0.05.

**Figure 2:**
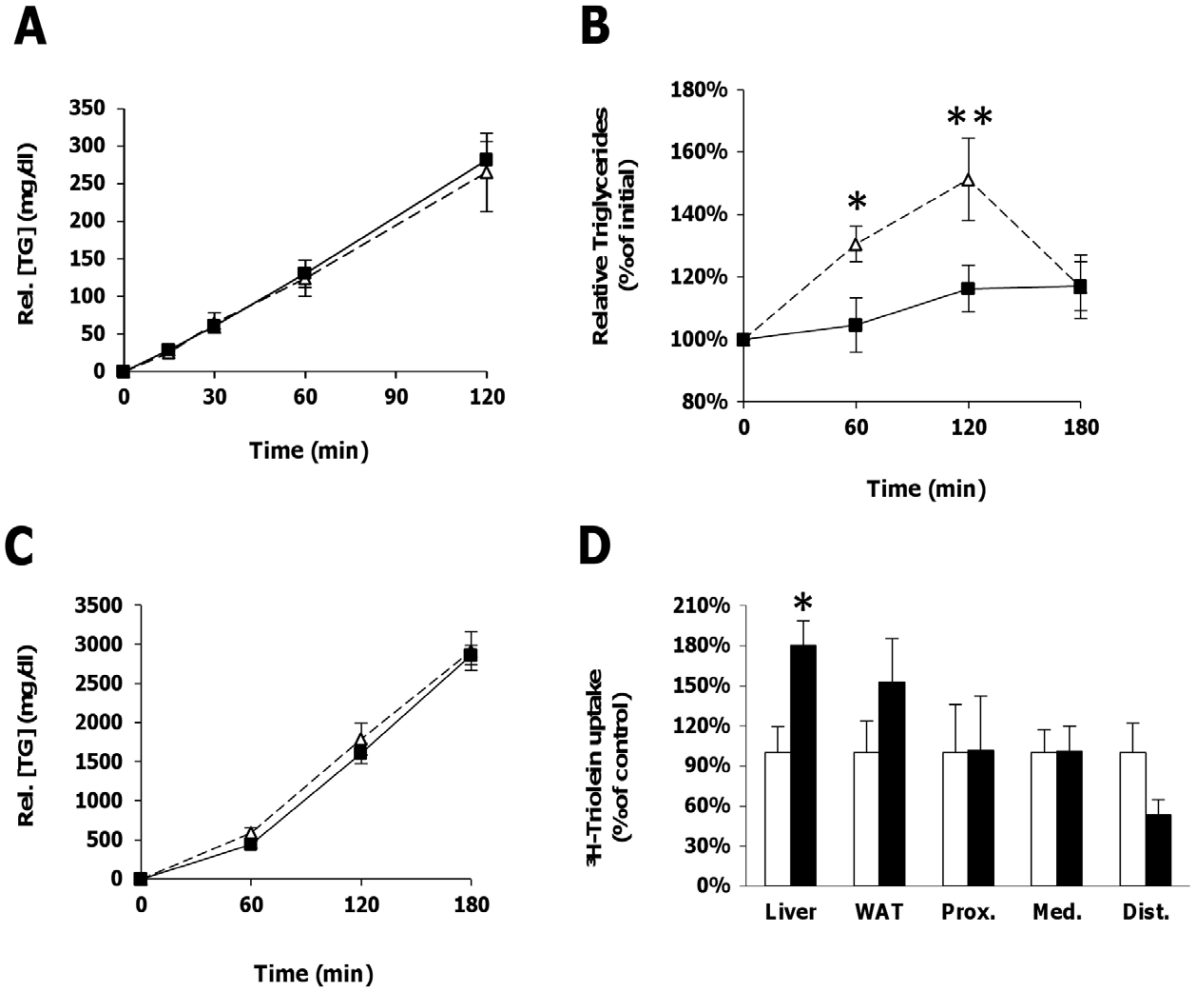
VLDL production, postprandial triglyceride response, intestinal lipid absorption and TRL clearance in apoE^−/−^ (Δ or □) and ApoE^−/−^LRP1^n2/n2^ (▪) mice. A–C, VLDL production after a Tyloxapol injection to inhibit lipolysis (A), postprandial triglyceride response after gastric olive oil load (B) and lipid absorption and chylomicron production after a combined gastric olive oil load and Tyloxapol injection (C) (n = 6–10 per genotype). D, Postprandial accumulation of ^3^H-Triolein in liver, white adipose tissue (WAT) and proximal (Prox.), medial (Med.) and distal (Dist.) intestine 2 h after a gastric load with olive oil mixed with ^3^H-Triolein (E) (n = 5 per genotype). Data are mean±SEM. *P<0.05, **P<0.001.

When crossing LRP1^n2/n2^ mice into an apoE^−/−^ background, the LRP1 NPxYxxL-motif inactivating mutation had no significant effect on total cholesterol levels in the absence of apoE (Figure 1A). Plasma triglyceride levels, however, were significantly 1.3-fold reduced in the apoE^−/−^LRP1^n2/n2^ mice compared to the apoE^−/−^ control mice (Figure 1A). Analysis of plasma apolipoprotein content revealed a decrease of apoB48 concentrations in apoE^−/−^LRP1^n2/n2^ mice (Figure 1B–C). While cholesterol distribution was similar between the two mouse genotypes (Figure 1D), triglyceride content was strongly decreased in the CR/VLDL fraction in apoE^−/−^LRP1^n2/n2^ mice when fasted (Figure 1F). Similar decreased triglyceride content in the CR/VLDL fractions was observed in the lipoprotein profile of apoE^−/−^LRP1^n2/n2^ mice 2 hours after receiving a fat load mimicking the postprandial state (Figure 1E & 1G). The decrease in TRLs seen for apoE^−/−^LRP1^n2/n2^ mice could not be linked to a reduction in hepatic VLDL-triglyceride production, as this was almost identical for both groups after Tyloxapol injection to inhibit lipolysis (Figure 2A). The postprandial triglyceride response after an oral fat load was, in contrast to control apoE^−/−^ mice, almost undetectable in apoE^−/−^LRP1^n2/n2^ mice (Figure 2B). When mice were given an oral fat load together with injection of Tyloxapol, we could see that apoE^−/−^LRP1^n2/n2^ and apoE^−/−^ mice had a similar postprandial triglyceride accumulation in the circulation (Figure 2C). These results exclude a possible contribution of both impaired lipid absorption and/or chylomicron production. To evaluate if accelerated clearance of TRLs could be a possible explanation, mice were given an oral load of a mixture of olive oil and a radioactive triglyceride, ^3^H-triolein, and sacrificed two hours later to harvest organs. Quantification revealed a significant 1.8-fold increase in the uptake of ^3^H-triolein in the liver for apoE^−/−^LRP1^n2/n2^ mice compared to apoE^−/−^ controls, while no significant differences were seen for adipose tissue and the small intestine (Figure 2D). The data suggest that apoE^−/−^LRP1^n2/n2^ mice have enhanced postprandial hepatic clearance of TRLs.

### Compensatory LDLR Up-regulation in ApoE^−/−^LRP1^n2/n2^ Mice is Associated with Increased Chylomicron Remnant Clearance

**Figure 3:**
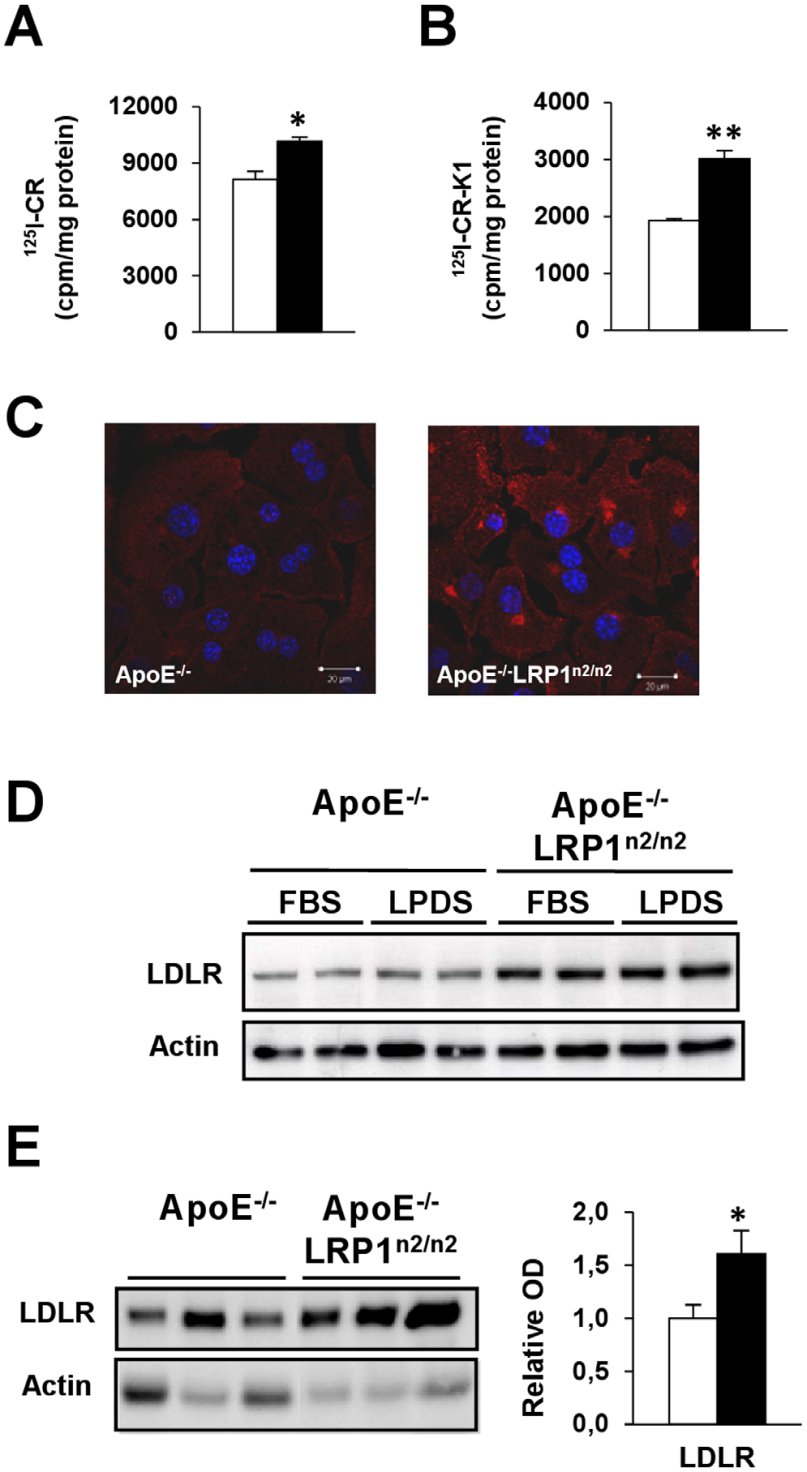
Compensatory LDLR up-regulation associated with an increased chylomicron remnant clearance. A–C, Internalization of ^125^I-CR (A) and ^125^I-CR-K1 (B) in primary hepatocytes and LDLR immunofluorescence staining in primary hepatocytes (bars are 20 µm) (C). D, Immunoblot analyses of hepatocytes for LDLR and β-actin protein levels after a 16 h incubation period with either 10% FBS or 10% Lipoprotein Deficient FBS (LPDS). E, Immunoblot analysis of microsomal liver extracts for LDLR and β-actin protein levels (n  = 6 per genotype). ApoE^−/−^ (□) and apoE^−/−^LRP1^n2/n2^ (▪) mice, data are mean±SEM. *P<0.05, **P<0.005.

In order to identify whether the increased postprandial lipid accumulation in the liver was due to an increased uptake of TRLs into hepatocytes, ^125^I-radiolabeled CR (Figure 3A) and CR supplemented with vitamin K1 (CR-K1) (Figure 3B) were evaluated *in vitro* for their uptake by primary hepatocytes. Uptake of radiolabeled CR and CR-K1 was respectively significantly 1.25-fold and 1.5-fold increased in hepatocytes isolated from the apoE^−/−^LRP1^n2/n2^ mice compared to the apoE^−/−^ control cells. A possible compensatory mechanism for CR clearance might be upregulated, as it was previously shown that the LRP1 mutation negatively affects the internalization property of the receptor [7] and that apoE-deficiency negatively affects LRP1 mediated clearance of CR-K1 in osteoblasts [33]. It is known that there exists a certain degree of functional redundancy between LDLR and LRP1 at the level of the liver [6]. Both via immunofluorescence (Figure 3C) and immunoblotting (Figure 3D–E) a significant increase in the expression levels of LDLR in hepatocytes (Figure 3C–D) and liver extracts (showing a 1.6-fold increase) (Figure 3E) could be observed between apoE^−/−^LRP1^n2/n2^ and apoE^−/−^ mice. These results suggest that LRP1 NPxYxxL-motif inactivation leads to a compensatory up-regulation of the LDLR, which could be responsible for improved CR clearance via hepatocytes.

### Inefficient Insulin-mediated LRP1 Translocation and Impaired Slow Recycling

**Figure 4:**
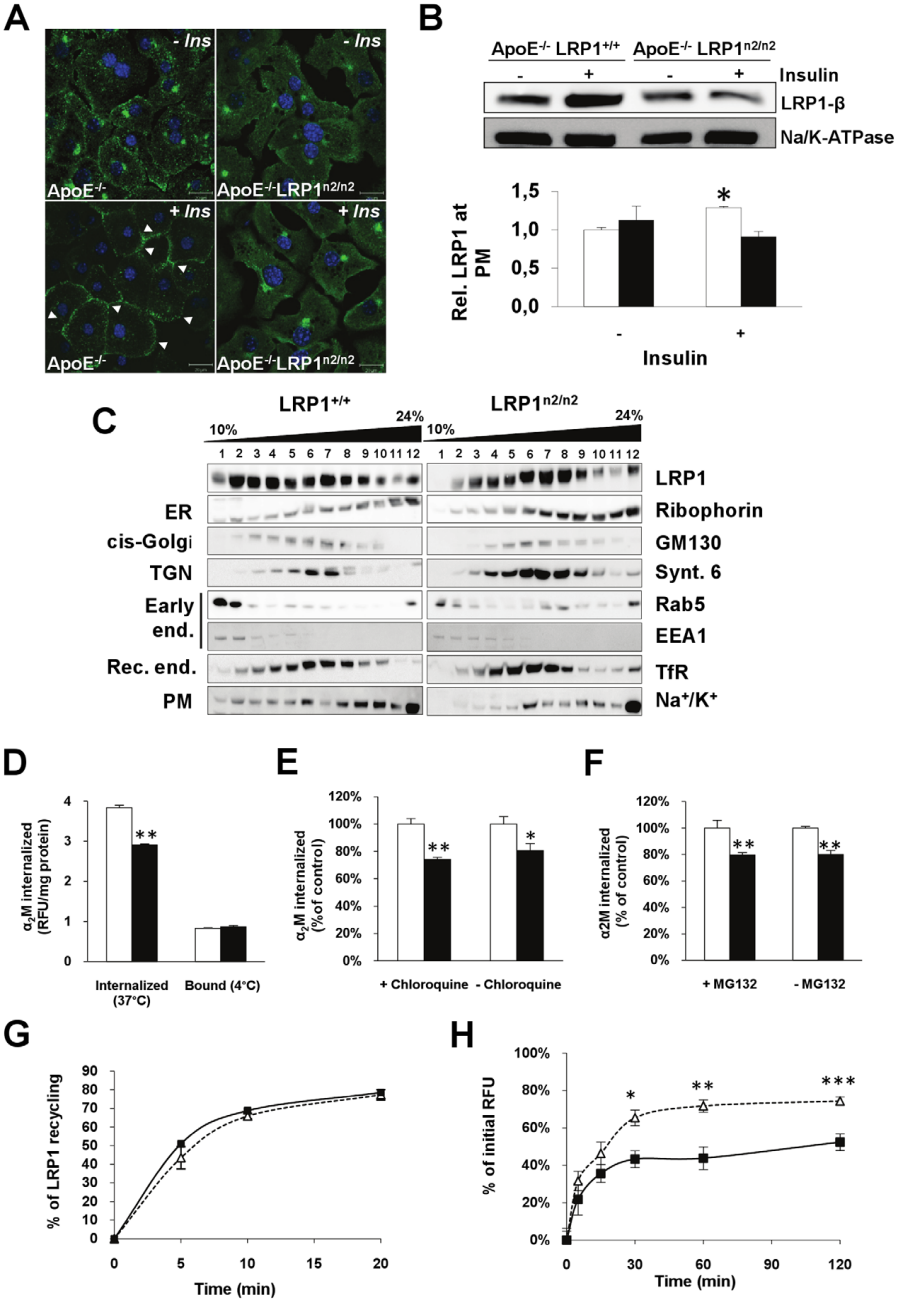
Inefficient insulin-mediated LRP1 translocation and impaired slow recycling. A–B, LRP1 staining in primary hepatocytes before and after insulin stimulation (A) and immunoblot analysis (B) in liver plasma membrane (PM) extracts [ApoE^−/−^ (□) and apoE^−/−^LRP1^n2/n2^ (▪)] before and after insulin injection (n = 3–4; bars are 20 µm). C, Cell Fractionation analysis of LRP1^+/+^ and LRP1^n2/n2^ MEFs. D–F, Steady-state internalization or binding (4°C) of FITC-α_2_M in mouse embryonic fibroblasts (MEFs) (D), steady-state internalization of FITC-α_2_M in MEFs in the absence (−) or presence (+) of either a lysosomal inhibitor, chloroquine (E), or a proteasomal inhibitor, MG123 (F) [twice in triplicate, LRP1^+/+^ (□) and LRP1^n2/n2^ (▪)].G–H, Fast (G) and slow (H) recycling kinetics of LRP1 in MEFs at the indicated time intervals [twice in triplicate, LRP1^+/+^ (□) and LRP1^n2/n2^ (▪)]. Data are mean±SEM. *P<0.05, **P<0.005, ***P<0.001.

We evaluated whether the mutation had an additional effect on insulin-mediated LRP1 translocation to the PM. In primary hepatocytes from apoE^−/−^ mice, LRP1 translocated to the PM upon insulin stimulation. In apoE^−/−^LRP1^n2/n2^ hepatocytes, however, LRP1 did not translocate efficiently (Figure 4A). Additionally, a different cellular distribution of LRP1 with a predominantly juxta-nuclear staining in the apoE^−/−^LRP1^n2/n2^ hepatocytes compared to apoE^−/−^ hepatocytes was observed. *In vivo*, absence of increased LRP1 at the PM after insulin injection was also found in preparations of purified PM isolated from apoE^−/−^LRP1^n2/n2^ livers (Figure 4B). Cell fractionation of wild-type LRP1 MEFs revealed that mature wild-type LRP1 (Figure 4C; LRP1) has a bimodal-distribution with the largest amounts of mature LRP1 present in the *cis*-Golgi to trans-golgi network (TGN) and the endosomal fractions [29]. Fractionation of MEFs with the LRP1 NPxYxxL mutation showed a unimodal distribution, as LRP1-β is not abundantly present in the endosomal fractions but the bulk of the protein is rather restricted to Golgi and recycling endosomal fractions (Figure 4C; LRP1-β). These distribution differences are suggestive for an altered cellular trafficking of LRP1 carrying the NPxYxxL inactivation. Initial binding to the plasma membrane of α_2_M was not different between LRP1^+/+^ and LRP1^n2/n2^ MEFs. The internalization rate, however, was significantly reduced by almost 25% (Figure 4D). Absence or presence of either lysosomal inhibitor chloroquine (Figure 4E), or the proteasomal inhibitor, MG132 (Figure 4F), did, however, not significantly improve the α_2_M internalization rate in LRP1^n2/n2^ MEFs. As LRP1 degradation was not significantly enhanced, we evaluated fast LRP1 recycling. After incubation with a fluorochrome labelled specific LRP1 antibody (Alexa488-5A6), the quantity of LRP1 recycling back to the cell surface was measured. LRP1 fast recycling kinetics in LRP1^+/+^ and LRP1^n2/n2^ MEFs were, however, almost identical (Figure 4G). Therefore, recycling from inside the cell to the plasma membrane at steady state was also investigated as described [35]. Cells were incubated with Alexa488-5A6 for 30 min, washed and chased for 2 h, to achieve a steady-state distribution. After this step, return of labelled LRP1 from perinuclear endosomal recycling compartments, slow recycling, was measured and revealed that 2 h after chase up to 75% of labelled wild-type LRP1 recycled back to surface, whereas only 50% of labelled LRP1 in the LRP1^n2/n2^ MEFs reached the cell surface (Figure 4H). These results indicate that the NPxYxxL motif is important for insulin-mediated translocation of LRP1 and slow perinuclear endosomal recycling.

### Reduced Atherosclerosis Development in ApoE^−/−^LRP1^n2/n2^ Mice

**Figure 5:**
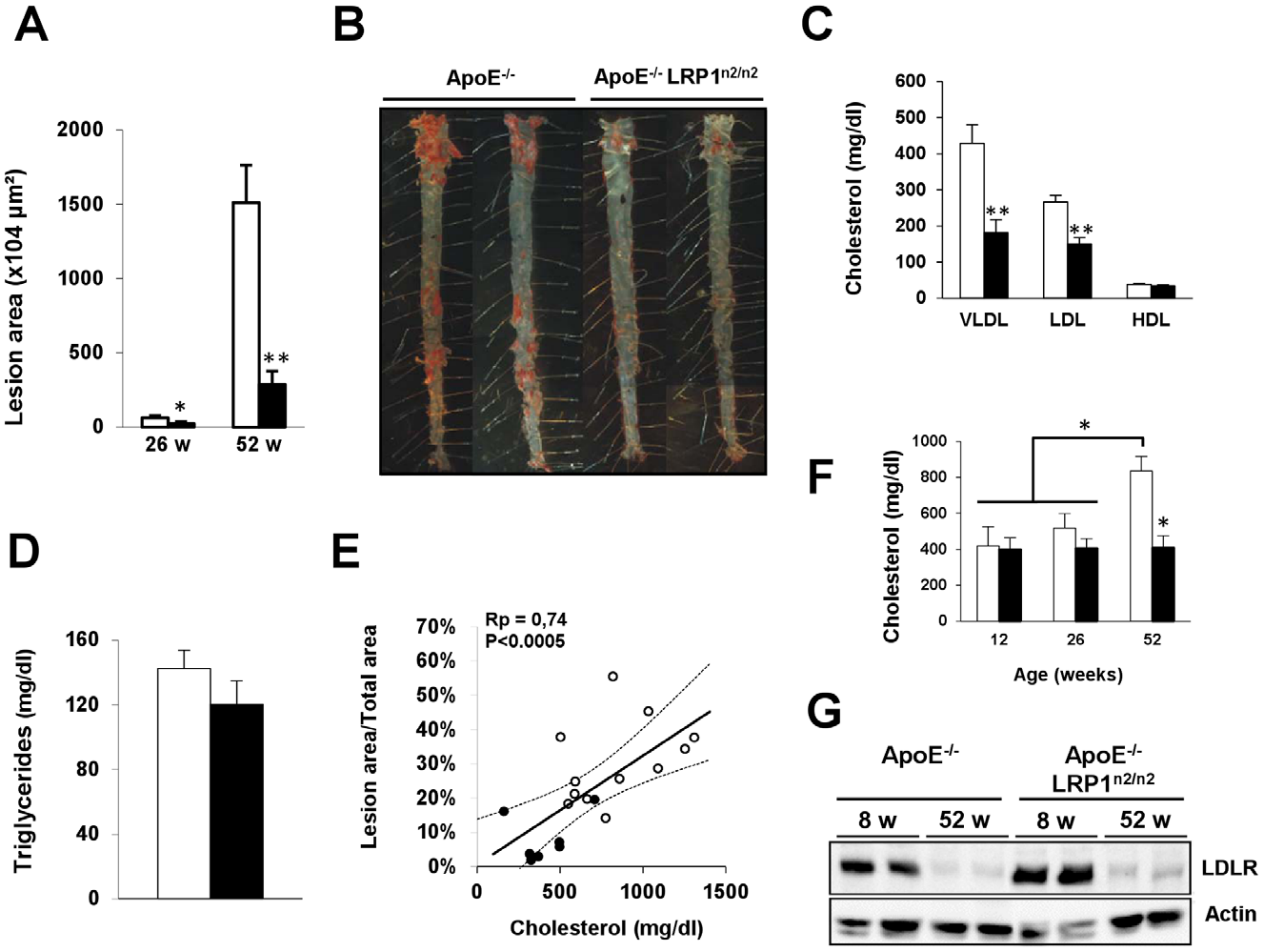
Reduced atherosclerosis development in ApoE^−/−^LRP1^n2/n2^ mice. A–B, Spontaneous atherosclerosis development in 26- (A) and 52-week (A–B) old mice. C–E, Different cholesterol levels in lipoprotein fractions (VLDL, LDL and HDL) separated via sequential ultracentrifugation in mice at 52-weeks of age (C), total triglyceride levels (D) and correlation plot between atherogenesis and total cholesterol for individual mice (E). Statistical analysis via determination of the Pearson’s correlation coefficient (Rp) revealed a significant positive correlation between atherosclerosis load in the aorta and circulating cholesterol levels. F, Total plasma cholesterol levels in mice at 12-, 26- or 52-weeks of age. G, Immunoblot analyses of hepatic LDLR and β-actin expression levels in 8- and 52-week old mice. ApoE^−/−^ (□ or ○) and apoE^−/−^LRP1^n2/n2^ (▪ or •) mice, n = 7–12 on a chow diet, data are mean±SEM. *P<0.005, **P<0.0005.

The impact of inefficient insulin-mediated translocation on spontaneous atherosclerosis development was evaluated in 26 week old mice. At this age, there was only minor development of early lesions. Nevertheless, apoE^−/−^LRP1^n2/n2^ mice had significantly less plaque load compared to apoE^−/−^ mice (2×n = 8; Figure 5A). For advanced plaques analysis mice were allowed to age up to 52 weeks. At this age, just 8% of the aortic surface of apoE^−/−^LRP1^n2/n2^ mice (n = 7) had Sudan IV positive lesions in comparison to 30% in the control apoE^−/−^ group (n = 12; Figure 5B). Analyzing plaque volume in ascending aortas confirmed that plaques in apoE^−/−^ mice (n = 6) were 1.6-fold larger compared to plaques in apoE^−/−^LRP1^n2/n2^ mice (n = 8) (data not shown). Plasma cholesterol levels in 52-weeks of age apoE^−/−^ mice were significantly 2-fold higher compared to apoE^−/−^LRP1^n2/n2^ mice (resp. 836±80 mg/dl vs. 411±66 mg/dl) due to higher VLDL- and LDL-cholesterol levels (Figure 5C). Assessment of the total TG levels indicated higher levels for the apoE^−/−^ mice compared to apoE^−/−^LRP1^n2/n2^ mice; however, not significantly (Figure 5D). There was a significant correlation between plaque load and cholesterol levels (Figure 5E). The observed higher cholesterol levels in apoE^−/−^mice of 52 weeks were age-dependent (Figure 5F) and associated with a significant age-dependent decrease in total hepatic LDLR levels (Figure 5G). Still despite this decrease, the LDLR expression levels of aged apoE^−/−^LRP1^n2/n2^ mice were 1.5-fold higher than aged-matched apoE^−/−^mice (data not shown). The data suggest that in the absence of apoE impaired LRP1 function correlates with improved atherosclerosis outcome.

## Discussion

The results presented here provide evidence for a protective role of impaired LRP1 translocation, which correlates with LDLR upregulation, on postprandial lipoprotein clearance and atherosclerosis. Even in an apoE-deficient model in which LRP1-mediated clearance of lipoprotein particles, now devoid of apoE, is impaired, further LRP1 dysfunction drives the system to compensate for this loss by upregulation of LDLR expression, especially at the liver. The clearance of postprandial triglyceride-rich remnants at the liver in the space of Disse is mediated via three receptor systems being syndecan 1, LRP1, and LDLR [37]. Current view on the clearance process put forward a three step mechanism in which entering TRLs are first sequestered by HSPG before being further lipolytically processed and subsequently cleared via the three receptor systems at the surface of hepatocytes. Both the sequestration via HSPG as the internalization of TRLs via HSPG and LRP1 are suggested to be primarily mediated through apoE [38], [39]. In the absence of fully functional apoE, the clearance of TRL-remnants relies heavily on the LDLR mediated pathway via binding to apoB100 and LPL-mediated binding to HSPGs and LRP1 [14]. This is similar to the situation in humans having *APOE2* homozygosity as this apoE isoform has reduced binding capacity for the LDLR, LRP1 and HSPGs [37].

Surprisingly, in the absence of apoE further impairment of LRP1-mediated clearance via inactivation of the NPxYxxL motif in the intracellular domain of LRP1 (LRP1-ICD) was associated with reduced triglyceride levels and reduction of apoB48 containing lipoprotein particles. Further looking into this, we have observed that this reduction in apoB48-lipoproteins was neither linked to a change in hepatic VLDL production, nor to intestinal lipid absorption and chylomicron production. However, apoE^−/−^LRP1^n2/n2^ mice showed an increased clearance rate of postprandial triglycerides and increased accumulation of CR-derived lipids in the liver. These observations were supported by the increased clearance rate of CR by primary hepatocytes *in vitro* and are suggestive for an accelerated hepatic clearance of TRLs in the apoE^−/−^LRP1^n2/n2^ mice. Rohlmann et al. were the first ones to show that hepatic LRP1 inactivation was associated with increased hepatic LDLR mRNA and protein expression [6]. In agreement with their observation, the inactivation of the NPxYxxL motif was associated with a significant increase of LDLR protein expression in hepatocytes. In our model, which relies as mentioned above on the LDLR-mediated clearance of TRLs, it is conceivable that this upregulated LDLR expression is responsible for the improved clearance of postprandial lipids observed. Both LDLR and LRP1 are regulated via SREBPs transcription factors, which are themselves regulated via intracellular cholesterol levels [40], [41]. Thus it is possible that at the liver the LRP1 knock-in mutation could influence intracellular cholesterol levels negatively, therefore leading to compensatory upregulation of hepatic LDLR expression. How LRP1 would influence intracellular cholesterol levels in hepatocytes in absence of its main ligand involved in lipoprotein clearance, apoE, was beyond the scope of this study and needs further investigation. However, this effect could be mediated via interaction of LRP1 with LPL and apoA-V present on lipoproteins [42], but could also be more complex as LRP1 is also involved in regulating hepatic ABCA1 expression and translocation and HDL secretion [19], [43].

Recently evidence was provided that hepatic LRP1 translocates to the plasma membrane after insulin stimulation, thereby enhancing ligand uptake capacity [11]. Our results indicate that inactivation of the LRP1 NPxYxxL motif abrogated this process. Impairment of this regulated postprandial increase of clearing capacity upon postprandial insulin stimulation could be a further explanation for the relatively strong compensatory upregulation of LDLR expression. Additionally, impaired translocation of mutant LRP1 was associated with alteration in cellular distribution. Inactivation of the NPxYxxL motif reduces the amount of LRP1 in the endosomal compartments in steady-state conditions in MEFs. A possible enhanced lysosomal or proteosomal degradation due to the NPxYxxL inactivation was not observed. It has been shown that a region of the LRP1 intracellular domain between amino acid 60 and 78, encompassing NPxYxxL, may contain an important motif contributing to LRP1 regulation by the proteasome [44]. However, the data suggest no relevant contribution of this pathway to the observed differences in post-endocytosis LRP1 trafficking between cellular compartments when inactivating the NPxYxxL motif. Effects of the LRP1 knock-in mutation on fast recycling efficiency was evaluated as it was shown in polarized cells using overexpression of truncated LRP1 mini-receptors that YxxL inactivation resulted in a non-polarized recycling phenotype as apical transcytosis was not completely prevented [45]. Yet, in our settings we have shown that fast LRP1 recycling in LRP1^n2/n2^ MEFs was identical to the wild-type control MEFs. In contrast, slow perinuclear recycling seemed to be impaired when inactivating the NPxYxxL motif, suggesting that this type of motifs is involved in slow recycling of receptors. This novel finding requires additional studies to determine whether the NPxY or YxxL motif is dominantly involved and to elucidate their exact mechanism and contribution in this receptor trafficking process. Slow endocytic recycling has been shown before to be insulin-responsive [46] and can therefore be an explanation for the impaired postprandial recruitment of mutant LRP1 to the plasma membrane.

LRP1 dysfunction is correlated with significant hepatic LDLR expression upregulation, although this is not associated with improved total cholesterol and also LDL-cholesterol levels in 12-week old apoE^−/−^LRP1^n2/n2^ mice compared to the apoE^−/−^ controls. It also did not significantly impacted LDL circulation time, as evaluation of systemic LDL clearance rates did not reveal any significant difference between the two genotypes (data not shown). Nevertheless, a potential contribution of the LRP1 NPxYxxL inactivation on the apoE-deficient background was evaluated for a possible effect of LRP1 on atherogenesis [47]. As observed, differences in spontaneous atherogenesis were minor but significantly smaller in 26-week old apoE^−/−^LRP1^n2/n2^ mice compared to apoE^−/−^ controls. Mice are more often in a postprandial state, thus it is conceivable that over time more rapid clearance of atherogenic remnant TRLs might be beneficial for atherogenesis. When mice were allowed to age for up to one year on a chow diet before evaluating atherosclerotic lesion development in the aorta, this difference was much more apparent. Surprisingly, one-year old apoE^−/−^LRP1^n2/n2^ mice also had significantly lower total cholesterol, VLDL-cholesterol and LDL-cholesterol levels compared to the apoE^−/−^ mice. This also translated as reduced atherogenesis in the mutant mice on the apoE-deficient background, which was significantly correlated with the individual cholesterol levels of the mice. It has been shown that upon aging the LDLR expression is significantly downregulated in mice and men [48], [49]. It is conceivable that at a certain point in their lifespan LDLR expression is getting critically low in apoE^−/−^ mice and therefore LDL-cholesterol levels will increase in the aging animal. As hepatic LDLR expression is upregulated in apoE^−/−^LRP1^n2/n2^ mice, they might take longer to reach a critical point of low LDLR expression or the critical low LDLR expression in peripheral tissue can be compensated by the increased hepatic LDLR expression. Although our results would support this hypothesis, we can only speak in terms of correlations and as such this will still require further investigation.

However, the fact that LRP1 impairment is associated with decreased atherosclerosis is the most striking finding is this study. Studies published thus far have demonstrated that LRP1 inactivation is correlated with deleterious effects on atherosclerosis [7], [18], [20], [21]. In particular these findings are in contrast to findings in our previous study where LRP1^n2/n2^ mice were crossed into an LDLR-deficient (LDLR^−/−^) background [7] and require additional studies. LDLR^−/−^LRP1^n2/n2^ showed increased atherogenesis, when put on a high fat/high cholesterol diet. Although we do not provide an explanation for this discrepancy in the current study, there are some obvious differences between both models that can support this contrast in atherosclerosis development. In the LDLR^−/−^LRP1^n2/n2^ mice accumulation of atherogenic remnant TRLs, both on a chow and on a high fat/high cholesterol diet, was observed, while the apoE^−/−^LRP1^n2/n2^ model, used in the current study, have significantly reduced remnant TRLs and LDL-cholesterol levels. Mainly, the two models are different in their lipoprotein handling and consequently in atherosclerosis development likely because of the absence versus presence of LDLR as discussed above. Furthermore, while in the previous study high fat/high cholesterol diet feeding produced mostly advanced lesions, the current settings of spontaneous atherosclerosis merely produced early lesions (data not shown). This might implicate that the reported roles for macrophage LRP1 in mediating apoptosis and efferocytosis do not yet have a great impact on atherogenesis and plaque progression [7], [20], [21]. It is also important to keep in mind that our model is a more complex situation, as LRP1 is dysfunctional in every tissue and cell type it is expressed without expression of apoE, one of its primary ligands [4]. This makes that phenomena observed in tissue specific knock-out models cannot always be directly extrapolated to observations made using our genetic approach. Moreover, the previously observed impaired apoE-mediated inhibition of platelet-derived growth factor-BB (PDGF-BB)-stimulated smooth muscle migration was relevant for increased plaque progression in LDLR^−/−^LRP1^n2/n2^ mice, but is certainly not applicable for the apoE-deficient model in the current study. This is yet another factor that might explain the contrasting effects seen for atherosclerosis development.

In summary, the obtained results show that in the absence of its role in the apoE-mediated catabolism of TRL remnants, due to apoE-deficiency, LRP1 dysfunction is improving the clearance of postprandial lipoproteins possibly via hepatic upregulation of LDLR expression. Moreover we could show that the NPxYxxL motif is important for the insulin-mediated translocation and endosomal sorting of LRP1. Despite its negligible impact on cholesterol levels in young mice, the compensatory LDLR upregulation became highly relevant for maintaining low cholesterol levels later on in life, which significantly correlated with reduced atherosclerosis development in old apoE^−/−^LRP1^n2/n2^ mice.

Altogether, our study presents experimental evidence *in vivo* suggesting that LRP1 dysfunction is likely not a secondary factor relevant for development of type III hyperlipidemia. However, we provide for the first time evidence that compromising LRP1 function has atheroprotective consequences in an apoE-deficient background, as previous studies only attributed atherogenic consequences to LRP1 inactivation [18]. This is especially relevant in a situation in which apoE binding properties to HSPGs, LDLR and LRP1 are compromised as in patients homozygous for the APOE2 isoform. Furthermore, our results support the hypothesis that *APOE2* homozygotes are hypocholesterolemic due to the upregulation of hepatic LDLR and consequently decreased LDL levels [14]. In this view our results emphasize the importance for further investigating the impact of targeting hepatic LRP1 function in the apoE-mediated catabolism of TRL remnants in relation to postprandial dyslipidemia and the other risk factors affecting atherosclerosis development.
